# Screening for ovarian cancer in women with varying levels of risk, using annual tests, results in high recall for repeat screening tests

**DOI:** 10.1186/1897-4287-9-11

**Published:** 2011-11-23

**Authors:** Marielle AE Nobbenhuis, Elizabeth Bancroft, Eleanor Moskovic, Fiona Lennard, Paul Pharoah, Ian Jacobs, Ann Ward, Desmond PJ Barton, Thomas EJ Ind, John H Shepherd, Jane E Bridges, Martin Gore, Chris Haracopos, Susan Shanley, Audrey Ardern-Jones, Sarah Thomas, Ros Eeles

**Affiliations:** 1 Department of Gynaecological Oncology, The Institute of Cancer Research & The Royal Marsden Foundation Trust, Cotswold Road and Fulham Road, SW3 6JJ, London, UK; 2 Section of Cancer Genetics, The Institute of Cancer Research & The Royal Marsden Foundation Trust, Cotswold Road and Fulham Road, SW3 6JJ, London, UK; 3 Department of Radiology, The Institute of Cancer Research & The Royal Marsden Foundation Trust, Cotswold Road and Fulham Road, SW3 6JJ, London, UK; 4 Cancer Research UK Human Cancer Genetics Group, Department of Oncology, University of Cambridge, Hills Road, CB2 0QQ, Cambridge, UK; 5 Department of Gynaecological Oncology, UCL, 1st Floor Maple House, 149 Tottenham Court Road, London W1T 7NF London UK

**Keywords:** ovarian cancer, screening, transvaginal ultrasound, serum CA125, *BRCA *gene mutation, bilateral salpingo-oophorectomy

## Abstract

**Background:**

We assessed ovarian cancer screening outcomes in women with a positive family history of ovarian cancer divided into a low-, moderate- or high-risk group for development of ovarian cancer.

**Methods:**

545 women with a positive family history of ovarian cancer referred to the Ovarian Screening Service at the Royal Marsden Hospital, London from January 2000- December 2008 were included. They were stratified into three risk-groups according to family history (high-, moderate- and low-risk) of developing ovarian cancer and offered annual serum CA 125 and transvaginal ultrasound screening. The high-risk group was offered genetic testing.

**Results:**

The median age at entry was 44 years. The number of women in the high, moderate and low-risk groups was 397, 112, and 36, respectively. During 2266 women years of follow-up two ovarian cancer cases were found: one advanced stage at her fourth annual screening, and one early stage at prophylactic bilateral salpingo-oophorectomy (BSO). Prophylactic BSO was performed in 138 women (25.3%). Forty-three women had an abnormal CA125, resulting in 59 repeat tests. The re-call rate in the high, moderate and low-risk group was 14%, 3% and 6%. Equivocal transvaginal ultrasound results required 108 recalls in 71 women. The re-call rate in the high, moderate, and low-risk group was 25%, 6% and 17%.

**Conclusion:**

No early stage ovarian cancer was picked up at annual screening and a significant number of re-calls for repeat screening tests was identified.

## Background

Primary ovarian cancer is the fourth most common cause of cancer-related death in the United Kingdom with 4500 deaths each year [1]. The identification of *BRCA1 *and *BRCA2 *gene mutations and their relationship to ovarian cancer has resulted in an awareness of family history in many women with ovarian cancer [2-5]. Several studies have investigated the role of screening in women with a positive family history of ovarian cancer. Unfortunately, no clear evidence of the efficacy of different screening strategies has been documented [6-8]. The normal life-time risk of developing epithelial ovarian cancer in the UK is estimated to be 1-1.5%. This risk is higher in women with a family history of ovarian cancer and increases with the number of affected relatives and the closeness of the relationship [9]. In women with a known *BRCA1 *or *BRCA2 *gene mutation the cumulative life-time risk of developing ovarian cancer can be as high as 60% and 27%, respectively, although it may be between 11-39% if the family history is less strong [4,10,11]. The prognosis of ovarian cancer is good when diagnosed at an early stage (FIGO stage I-II) with 80--95% 5-year survival. However, the majority of ovarian cancer cases (70%) are detected at an advanced stage with poor survival [12].

For women at high-risk of developing ovarian cancer, management options still include a) ovarian cancer screening by CA125 measurement and or transvaginal ultrasound, with the intention to reduce mortality by detecting ovarian cancer at an early stage, or b) prophylactic bilateral salpingo-oophorectomy (BSO). The optimal management policy in women with moderate or low-risk of developing ovarian cancer remains unclear.

In this study, we assessed ovarian cancer screening outcomes in women with a positive family history of ovarian cancer stratified in a low, moderate or high-risk group of developing ovarian cancer. We defined the number of ovarian cancer cases identified, the number of re-calls because of abnormal test results, and present the characteristics of women who underwent prophylactic BSO.

## Methods

We conducted a retrospective audit of 545 women consecutively referred because of a positive family history of ovarian cancer from January 2000 to December 2008 to the Cancer Genetics Clinic at The Royal Marsden NHS Foundation Trust in London, UK, for consideration of annual screening. At the initial clinic visit the family history was documented, assessed and confirmed in a standardised format. Three risk categories were identified. Women fulfilling the family history criteria shown in Table 1 (NICE Guidelines on Familial Breast Cancer 2004), with an expected life-time risk of ovarian cancer of greater than 10% were considered to be in the high-risk group [13]. Women not meeting these criteria were stratified either into the moderate-risk group (when life-time risk = 4-10%), or in the low-risk group when there was no significant family history of ovarian cancer. Screening consisted of annual serum CA125 level measurements and a transvaginal ultrasound. All scanning was performed by one consultant radiologist (EM) with many years of transvaginal ultrasound experience. Screening results were interpreted and acted on by clinicians involved with no defined protocol other than using the risk of malignancy index (RMI) only. Serum CA125 levels above 35 kU/L were considered abnormal. Abnormal findings in at least one of the screening tests prompted repeat testing within 3 months. Adjuvant imaging (i.e. MRI) was organised urgently when malignancy was suspected and, if indicated, a diagnostic surgical procedure was performed (laparoscopy or laparotomy). Women in the high-risk group were offered genetic testing if possible (only if the affected relative could be tested or if there were no living affected relatives a founder mutation test could be found if she was from a relevant ethnic group). Women in the moderate-risk group were offered genetic testing only if indicated (e.g. if they were from a founder group where testing indicated even at moderate risk for ovarian cancer). Prophylactic BSO was offered to all high-risk women when they completed their family or reached the age of 40 years. During this procedure an endometrial sampling was taken, as this is standard practice in our centre.

**Table 1:** Increased risk criteria in women with a family history of breast and ovarian cancer [13]

Criteria	
1	Family contains 2 or more individuals with ovarian cancer who are first degree relatives
2	Family contains 1 individual with ovarian cancer and 1 individual with breast cancer diagnosed < 50 years who are first degree relatives
3	Family contains 1 individual with ovarian cancer and 2 individuals with breast cancer diagnosed < 60 years who are connected by first degree relationships
4	Family contains an affected individual with a mutation in one of the known ovarian cancer predisposition genes *BRCA1, BRCA2, MSH2, MSH6, MLH1, PMS1 *or *PMS2*
5	Family contains 3 individuals with colorectal cancer with at least 1 case diagnosed before 50 years and 1 case of ovarian cancer. All individuals are connected by first degree relationships
6	Criteria 1,2 and 3 can be modified where paternal transmission is occurring, i.e. families where affected relatives are related by second degree through an unaffected intervening male relative and a sister is affected are eligible

The provision of ovarian screening was discontinued at The Royal Marsden NHS Foundation Trust in 2004 as screening was concentrated in other centres, if appropriate; pending the results of studies of screening in high-risk families (the UKFOCSS study). All patients had a full family history taken out to third degree relatives and their risk was reviewed by a cancer geneticist (RE). Those patients who had been assessed as being at high-risk for ovarian cancer were offered participation in the UKFOCSS study (UK Familial Ovarian Cancer Screening Study). Those who were been assessed as being at moderate-risk were referred to St Georges Hospital, London, one of the very few NHS Gynaecology Departments which still offered ovarian cancer screening outside a research study to moderate-risk women, or to a local hospital if this was more convenient for the woman. All other patients were considered low-risk, indicating that some women formerly considered at increased risk were reclassified as low-risk and advised that there was no indication for further screening and were discharged. From 2004, new patients were still initially assessed in our clinic but referred according to risk group. Inclusion in the study ended in December 2008. The total number of prophylactic BSO was recorded as were the number of repeat CA125 measurements and transvaginal ultrasound visits due to abnormal findings. The expected number of ovarian cancers to be detected in this study was calculated, divided into women years tested by risk category and age group (using incidence rates of Antoniou *et al*, 2003) [4] Statistical analysis was performed using SPSS software (version 17.0, SPSS Inc. Chicago).

As this was a clinical audit, a formal ethical review was not required. According to hospital protocol the study was reviewed and approved by the Royal Marsden Clinical Audit Committee.

## Results

A total of 545 women were included in this study and all had at least one CA125 measurement and/or transvaginal ultrasound at baseline. The median age at entry was 44 years old (range 25-74 years), with the majority of women in the age group of 35 to 49 years old (61.3%) (Table 2). Most women had at least one child (370 out of 545; 67.9%). The total range was 0-7 children with a median of 2 children.

**Table 2:** Patient characteristics at baseline with results of genetic testing in the high-risk group

Patient characteristics		N	%	Genetic testing	N	%
**Age group**	< 35 years	42	7.7			
	35-49 years	334	61.3			
	≥ 50 years	169	31.0			
						
**Parity**	0	133	24.4			
	≥ 1	370	67.9			
	Unknown	42	7.7			
						
**Risk group**	High-risk	397	72.8	Mutation present	106	26.7
				Negative	34	8.6
				Not tested	257	64.7
						
	Moderate-risk	112	20.6	NA*		
	Low-risk	36	6.6	NA*		

*NA = not applicable

At baseline, the majority of women was in the high-risk group n = 397 (73%). The moderate- and low-risk group consisted of 112 (21%) and 36 (6.6%) women, respectively. In the high-risk group 140 women (35.3%) underwent genetic testing and in 106 (76%) a genetic mutation was identified. Among these women, 58 (55%) women were *BRCA1 *gene mutation carriers and 45 (43%) women had a *BRCA2 *gene mutation. In 2 (1.9%) an *MLH1 *gene defect mutation was identified. One (0.9%) woman was diagnosed with both a *BRCA1 *and *BRCA 2 *gene mutation. This 69 year old lady underwent a hysteroscopy, dilatation and curettage for an endometrial polyp which revealed a grade 3 endometrioid adenocarcinoma of the endometrium. She underwent a laparoscopic hysterectomy and bilateral salpingo-oophorectomy showing FIGO stage 1B endometrial cancer. Her ovaries and fallopian tubes were histologically normal.

In 2 women, epithelial ovarian cancer was diagnosed. The first case, a 50-year-old *BRCA1 *gene mutation carrier, presented at her fourth annual screening visit with an elevated CA125 of 3874 kU/L and bilateral complex ovarian masses with ascites on transvaginal ultrasound. She was asymptomatic. Her previous screening CA125 levels and transvaginal ultrasounds did not show any abnormalities. At her third annual screening visit, 14 months before she was diagnosed with ovarian cancer, her CA125 was 8 kU/L She underwent optimal debulking surgery and the final histopathology revealed Stage 3C poorly differentiated serous papillary adenocarcinoma of the ovary. The second ovarian cancer case was found in a 40 year old *BRCA1 *gene mutation carrier. She underwent a routine prophylactic BSO. The adnexa were grossly normal but the histology revealed Stage 1A poorly differentiated serous adenocarcinoma of the ovary. Her preoperative CA125 was 30 kU/L and no abnormalities were seen on transvaginal ultrasound. She had been undergoing annual screening for 5 years. Both patients are still alive after 6 and 4 years of follow-up, respectively.

Prophylactic BSO was performed in 138 women (25%). In most cases peritoneal washings and an endometrial sample were taken at the same time. Table 3 shows the characteristics of women who underwent a prophylactic BSO. The majority of women came from the high-risk group (94%); 69 (53%) of whom had tested positive for a gene mutation. None of the 8 moderate-risk women had had genetic testing and in one of these women ovarian stromal hyperplasia was found on histopathology. In the high-risk group 3 women (2.4%) had an ovarian/tubal abnormality showing cancer (Stage 1A, see above), tubal atypia and struma ovarii, respectively. In 2 women an endometrial abnormality was found; atypical hyperplasia and hyperplasia, on hysterectomy and curettage, respectively.

**Table 3:** Characteristics of 138 women who underwent prophylactic bilateral salpingo-oophorectomy

Risk Group	N	%	Genetic testing	N	%	Histopathology	N	%
**Moderate**	8	6.5	Not tested	8	100	NED	7	87.5
						Ovarian stromal hyperplasia	1	12.5
**High**	130	94.2	Not tested	60	46.2			
			Negative	1	0.8			
			*BRCA1 *mutation	37	28.5			
			*BRCA2 *mutation	30	23.0			
			*MLH1 *mutation	2	1.5			
						NED	125	96.0
						Cancer	1	0.8
						Atypia tube	1	0.8
						Struma Ovarii	1	0.8
						Atypical hyperplasia*	1	0.8
						Hyperplasia*	1	0.8

NED = no evidence of disease; * endometrium

A total of 2266 women years of follow-up were identified. The majority of women years (1623 women years) were in the high-risk group, with 522 and 121 women years in the moderate- and low-risk group, respectively. A total of 3.89 (95% C.I. 0.22-7.22) ovarian cancer cases were expected in the study population with 3.11, 0.68 and 0.11 cases in the high-, moderate-, and low-risk groups, respectively.

Table 4 shows the number of abnormal test results in our cohort. In the high-risk group 37 out of 397 (9%) of women had at least one elevated CA125 measurement and 65 (16%) had at least one abnormal transvaginal ultrasound scan during follow-up. In the moderate-risk group these figures were 4% in both groups, and in the low-risk group 3% and 6%, respectively. Equivocal transvaginal ultrasound results prompted a total of 108 re-calls in 71 women, with a majority of re-calls in the high-risk group. The median age of women needing a re-call transvaginal ultrasound was 43 years (range 29-68). In only 7 high-risk women these abnormal transvaginal ultrasounds resulted in a prophylactic BSO. In all these cases the final histopathology was normal. In all other cases repeat transvaginal ultrasounds returned to normal.

**Table 4:** Number of patients with abnormal ovarian screening test results and number of repeat tests among different risk groups

Risk group (Total no. pts)	No of pts with abnormal test result		No of repeat tests		Prophylactic BSO because of abnormal test	
	CA125 (%)	TVUS (%)	CA125 (%)	TVUS (%)	CA125	TVUS
High risk (397)	37* (9)	65* (16)	54 (14)	98 (25)	-	7
Moderate risk (112)	5 (4)	4 (4)	3** (3)	4 (6)	1	-
Low risk (36)	1 (3)	2 (6)	2 (6)	6 (17)	-	-

TVUS = transvaginal ultrasound; * includes 1 patient diagnosed with advanced ovarian cancer; ** no repeat tests in 2 patients: 1 patient with recurrent progressive breast cancer and 1 pregnant patient

Forty-three women had an abnormal CA125 measurement, resulting in a total of 59 repeat tests. The majority of elevated CA125 was seen in the high-risk group. Only one moderate-risk woman underwent a prophylactic BSO because of 2 consecutive elevated CA125 measurements (CA125 was 64 and 57 kU/l, respectively). Her transvaginal ultrasounds were normal. No abnormalities were found on final histopathology. In all, but three women, CA125 results came back to normal. Among these three women, one was diagnosed with advanced ovarian cancer, one woman was pregnant and the third diagnosed with recurrent breast cancer.

## Discussion

In this retrospective study we assessed ovarian cancer screening outcomes in 545 women with a positive history of ovarian cancer stratified into a low, moderate, or high-risk group of developing ovarian cancer.

A total of 2 (0.4%) ovarian cancer cases were identified. None of them were found during screening. Most studies looking at the effectiveness of ovarian cancer screening found a higher incidence of ovarian cases of about 1-3% [7,14]. Although our number of identified ovarian cancer cases was low in comparison with these studies, the annual screening detected the number of cases expected for a population of this age, risk spectrum and duration of follow-up. One reason for the low incidence of ovarian cancer is that our cohort included moderate- and low-risk women. Laframboise et al. reported 1 ovarian cancer in a series of 311 women of which only 10% were *BRCA *gene mutation carriers [15].

In our high-risk group 35% underwent genetic testing and in 76% a genetic mutation was identified. The reason for this high percentage could be the high proportion of Ashkenazi women (30%) and women with a previous history of breast cancer (47%) in this group.

Prophylactic BSO was performed in 25% of women and in one case early stage ovarian cancer was found, but on histology only. She had normal pre-operative screening results. Transvaginal ultrasound and CA125 measurements as screening tests are known to have their limitations. High false-positive rates have been identified, especially in pre-menopausal women, resulting in a high number of re-call visits, anxiety through equivocal test results, and even, unnecessary surgical intervention [14-16]. In our study we found at least one elevated CA125 in 43 women (8%) and at least one abnormal transvaginal ultrasound in 71 women (13%). In the majority of cases the repeat tests showed normal results, although in 8 women a BSO was performed due to a persistently abnormal test result. No ovarian cancer was found in these women. Other studies have shown a significant number of abnormal CA125 results and transvaginal ultrasounds during surveillance [15]. Oei et al. screened 512 high-risk women and found abnormalities in 21% of all transvaginal ultrasounds and in 4% of all CA125 measurements [17]. The majority of these abnormal results disappeared within 3-6 months, resulting in a high number of unnecessary re-calls. Meeuwissen et al. observed abnormal results in 19% of 383 high-risk women, with 64% of them returning to normal spontaneously [18].

The limitation of our study is that it has not been conducted in the form of a robust prospective protocol, but was an analysis of the clinical management guidelines at the time. Screening results were interpreted and acted on by clinicians involved with no defined protocol other than using the RMI only. Our study revealed a significant number of re-calls for repeat screening tests. No early stage ovarian cancer was identified at annual screening by serum CA125 and/or transvaginal ultrasound. Of the two ovarian cases found, one early stage ovarian cancer was found at prophylactic BSO. The other case was found during screening and presented with an advanced ovarian cancer. Both patients had been under surveillance for more than 4 years and had no abnormal results in their previous annual screening rounds. Several studies have already reported that annual screening of high-risk women will not reduce mortality from ovarian cancer [6,19]. A large study of 3532 high-risk women screened annually with transvaginal ultrasound and CA125 did not show any benefit in survival; the majority of ovarian cancer cases was found in an advanced stage [7]. Another study including 241 *BRCA *positive women who underwent ovarian surveillance concluded low sensitivity and positive predictive values of ovarian screening modalities and no additional benefit from screening [8].

More sophisticated screening criteria have been developed since the start of our study and we are awaiting the results of a large UK multi-centre trial, the UKFOCCS trial to clarify the role of different algorithms of ovarian screening in women with an increased genetic risk to assess if this improves upon the annual re-call algorithm. Since no effective screening programme has yet been identified, prophylactic BSO should be advised to all high-risk women after they completed their family which will reduce the risk of ovarian cancer associated with *BRCA*1 or *BRCA*2 mutation by 70-96% [20-22].

In conclusion, this study assessed the role of annual ovarian screening in women with a family history of ovarian cancer stratified into 3 different risk-groups. No early stage ovarian cancer was picked up at annual screening. This approach to screening led to a significant number of re-calls for repeat screening tests.

## Competing interests

The authors declare that they have no competing interests.

## Authors' contributions

RE and MG conceived the study, participated in the design of the study, collected data, and interpreted the results. EB, EM, FL, IJ, AW, DPJB, TEJI, JHS, JEB, CH, SS, AA-J, ST, collected data and interpreted the results. PP interpreted the results and performed the statistical analysis. MN collected and interpreted the data and performed the statistical analysis. All authors have been involved in drafting and/or revising the manuscript. All authors read and approved the final manuscript.
